# Reliability and Validity of the Spanish Version of the Brief-BESTest in Stroke Patients

**DOI:** 10.3390/jcm13102873

**Published:** 2024-05-13

**Authors:** Beatriz Hernández-Moreda, Inés Llamas-Ramos, Rocío Llamas-Ramos, Juan Luis Sánchez-González, Beatriz María Bermejo-Gil, Fátima Pérez-Robledo, Elisa Frutos-Bernal, Ana María Martín-Nogueras

**Affiliations:** 1 Department of Nursing and Physiotherapy, Faculty of Nursing and Physiotherapy, Universidad de Salamanca, 37007 Salamanca, Spain; beatrizhernandez@usal.es (B.H.-M.); rociollamas@usal.es (R.L.-R.); juanluissanchez@usal.es (J.L.S.-G.); beatriz.bermejo@usal.es (B.M.B.-G.); fatima_pr@usal.es (F.P.-R.); anamar@usal.es (A.M.M.-N.); 2 Instituto de Investigación Biomédica de Salamanca (IBSAL), 37007 Salamanca, Spain; 3 University Hospital of Salamanca, 37007 Salamanca, Spain; 4 Department of Statistics, Facultad de Medicina, Universidad de Salamanca, Campus Miguel de Unamuno, 37007 Salamanca, Spain; efb@usal.es

**Keywords:** Brief-BESTest, assessment, acquired brain injury, balance, physiotherapy

## Abstract

**Background**: Balance disorders and postural control treatments play an important role in fall prevention. The Brief-BESTest is a short-scale employed to evaluate balance and fall risk in different populations. Balance assessment is a fundamental element in patients with Acquired Brain Injury rehabilitation since postural alteration is one of the most frequent sequelae. The objective was to validate the Spanish version of the Brief-BESTest questionnaire in the stroke population. **Methods**: Subjects of both sexes aged over 18 years with a diagnosis of acute/chronic stroke were included. The BESTest, Mini-BESTest, Brief-BESTest, Berg Balance Scale, and Timed Up & Go Test were used to assess balance. The scales were implemented once. Cronbach’s alpha coefficient was used to assess the internal consistency and confirmatory factorial analysis was employed to assess validity. **Results**: A total of 44 patients with a mean age of 65.35 years (SD = 10.665) participated. Cronbach’s alpha coefficient showed a high internal consistency with a value of 0.839. In the criterion validity, there was a high positive correlation between the Brief-BESTest and BESTest (r = 0.879), Mini-BESTest (r = 0.808), and Berg Balance Scale (r = 0.711). **Conclusion**: The Spanish version of the Brief-BESTest scale is valid and reliable, showing adequate psychometric properties for balance assessment in patients with acute or chronic stroke.

## 1. Introduction

Acquired Brain Injury (ABI) is the third cause of death and the first cause of disability in adults [1] with an annual incidence of 104,701 new cases in Spain [2]. The most common cause is stroke, becoming a major social and economic problem. According to the Iberictus study, between 80,000 and 90,000 strokes occur annually in Spain, representing the second cause of death [3]. Fifteen percent of patients who suffer a stroke die, and the 30% of survivors remain in a situation of functional dependence with mobility problems in their activities of daily living. These mobility problems are caused due to hypertonia, spasticity, or rigidity [4] and balance disorders leading to an increased risk of falls [5].

Balance disorders and postural control treatments play an important role in fall prevention. Postural control is understood as the coordination of movement strategies to orient the body in space and stabilize the body’s center of mass during activities or stability disturbances [6]. It is a complex skill based on the interaction of the somatosensory, vestibular, visual, and cognitive systems [7]. Consequently, different balance assessment tools have been developed. Specifically, there are six balanced assessment systems: biomechanical constraints, stability/verticality limits, anticipatory postural adjustments, reactive postural control, sensory orientation, and dynamic gait.

Although it is already known that balance is affected by these systems, in the clinic assessments one or more functional tasks are evaluated regardless of which postural control system is affected. However, it is necessary to know which subsystem is responsible for the balance deficit to understand the cause and determine the most appropriate treatment program. In 2009, Fay Horak developed the Balance Evaluation Systems Test (BESTest) with 27 items encompassed in the six evaluation systems [8]. Due to the redundant nature of the components and the lengthy BESTest application process, the Mini-BESTest was developed with 14 items but only four of the six balance assessment systems are represented [9]. In 2012, Parminder used Rasch’s model and created a new version, the Brief-BESTest [10]. The Brief-BESTest is the more abbreviated and alternative version to the BESTest and Mini-BESTest. It is a shorter scale than the other two; therefore, less time is needed to administer it, namely less than 10 min and it represents the six dimensions based on the theory of the original BESTest. This aspect is very relevant in neurological patients due to the fatigue they present, so scales with a shorter application time are more accepted and suitable for these patients.

The Brief-BESTest has been shown to have good reliability and validity in examining the balance of individuals with chronic stroke [11], patients with chronic obstructive pulmonary disease [12] and even predicting falls in people with Parkinson’s disease [13]. Currently, the original Brief-BESTest has only been validated in the Turkish language [14].

Clinicians require quick, easy-to-use scales that are practical for assessing balance, fall risk, and selecting an appropriate rehabilitation plan. In clinical practice, having these resources available in Spanish will be very helpful. Therefore, the objective of this work was to validate the Brief-BESTest questionnaire in Spanish and to know its psychometric properties to have a simple, useful, and standardized tool for the functional assessment of stroke patients.

## 2. Materials and Methods

### 2.1. Design and Setting

A cross-sectional observational study was conducted. The sessions were implemented in the Faculty of Nursing and Physiotherapy of the University of Salamanca. It has been registered in ClinicalTrial.gov in December 2020 with the identifier NCT04673539 and has the approval of the Ethics Committee of the University of Salamanca with the registration number 585.

### 2.2. Participants

Subjects of both sexes aged over 18 years with a diagnosis of acute or chronic stroke were included. They voluntarily agreed to participate and signed an informed consent form and participated. All of them were recruited from the University Hospital of Salamanca, Association of Acquired Brain Injury of Salamanca and Kinhermo clinic between July 2022 and May 2023.

As exclusion criteria, those with cognitive impairment that prevented them from understanding commands, impaired balance due to other pathologies than stroke, unable to read or write, unable to walk more than 10 m independently, severe hearing loss or blindness, and not understanding of Spanish language were excluded.

### 2.3. Variables and Instruments

The following information was recorded in a medical record: date of birth, date of injury, marital status (single, married, or divorced), sex, dominant side (right-handed or left-handed), affected side (right or left), employment status/profession and level of education (primary, secondary, high school, vocational training, or university).

The main variable of this study was balance. It was assessed with the following scales or questionnaires: BESTest, Mini-BESTest, Brief-BESTest, Berg Balance Scale, and Timed Up & Go Test.

#### 2.3.1. BESTest or Balance Evaluation Systems Test

It is a balance assessment and training scale developed by Fay Horak in 2009 [8]. The BESTest consists of 36 items, encompassing 27 items grouped into six sections, and each item is scored from 0 to 3 (0 is the lowest level of functionality and 3 is the highest level of functionality); so that some of the items have several answers depending on whether the right or left limb is used. The test requires an administration time of 40 min.

#### 2.3.2. Mini-BESTest

It was proposed in 2010 by Franchignoni et al. [9] to be shorter and more accessible and feasible to use at the clinical level. It consists of 14 items encompassed in only 4 of the original 6 sections of the BESTest. The score range for each item is from 0 to 2 (0 being the lowest level of functionality and 2 being the highest level of functionality) for a total of 28 points and an application time of approximately 10–15 min.

#### 2.3.3. Brief-BESTest

It was designed in 2012 by Padgett et al. [10]. It contains 1 item from each section of the original BESTest; therefore, it consists of 6 items in total. It adds up to a total score of 24 points. It takes 8 to 10 min to administer depending on the patient.

#### 2.3.4. Berg Balance Scale (BBS)

It was created in 1989 by Katherine Berg [15] to assess balance ability in older people. It consists of 14 mobility tasks of varying degrees of difficulty. The tasks are divided into 3 domains: sitting balance, standing balance, and dynamic balance. Each exercise is scored on an ordinal scale from 0 to 4 with a maximum score of 56 points. A score of 0 means that the subject is unable to perform the task and a score of 4 means that the subject can perform the task correctly and independently. It requires an administration time of 10–15 min.

#### 2.3.5. Timed up & Go Test

Its first use was in 1991 [16]. It consists of measuring the time it takes the patient to get up from a chair, walk 3 m at a comfortable pace, turn around, return to the chair, and sit down with the back fully against the backrest. It requires an administration time of 3 min.

### 2.4. Procedure

Two physiotherapists conducted training to implement the scales. One led the session with the participant and the other recorded the results according to her judgment. In case of doubt, this was resolved by consensus. Once the patients had been recruited, the balance was assessed with the mentioned measuring instruments.

All the sessions were carried out in the same order; the physiotherapists introduced the patient to the object of this study and after completing the informed consent form and filling in their clinical history, the scales were implemented once the participant and the physiotherapists were ready. The scales were always implemented in the same order and only once: BESTest, Mini-BESTest, Brief-BESTest, Berg Balance Scale, and Timed Up & Go Test.

It has not been necessary to perform cross-cultural validation in this study because it has been previously performed in the validation of the BESTest and Mini-BESTest scales [17].

### 2.5. Statistical Analysis

Data for continuous variables are shown as mean ± standard deviation and categorical variables are shown as number and percentage.

Cronbach alpha coefficient was used to assess internal consistency. The Spearman correlation coefficient was calculated to evaluate criteria validity. To measure construct validity, a confirmatory factor analysis (CFA) was carried out. Given the nominal nature of the data, the maximum likelihood (ML) estimation method was used. To assess the goodness-of-fit of the model, the correction of the S-B$\chi^{2}$ on the degrees of freedom (S-B$\chi^{2}/gl$) is less than 3; the Tucker Lewis Index (TLI), the Normed Fit Index (NFI), and the Comparative Fit Index (CFI) (all incremental fit indices) if higher than the 0.90 are considered an adequate fit. The Root Mean Square Error of Approximation (RMSEA) must be lower than 0.08 for an optimal fit level.

The receiver-operating characteristics curve (ROC) was used to determine the cut-off point on the Brief-BESTest scale for the risk of falls. The area under the curve (AUC), sensitivity, and specificity values were obtained from the ROC analysis. AUC values were interpreted according to Hosmer and Lemeshow (AUC ≥ 0.9: outstanding discrimination, AUC = 0.8–0.9: excellent discrimination; AUC = 0.7–0.8: acceptable discrimination) [18].

The skewness coefficient was used to assess the floor effect and the ceiling effect of the Brief-BESTest. The percentage of participants scoring in the top 10% (i.e., total score > 21) or the bottom 10% (i.e., total score < 3) was also taken into account [19]. A proportion of participants above 20% was considered to be a substantial ceiling effect, respectively, floor effect [20].

For the analyses previously indicated, SPSS Statistics version 28.0 and SPSS Amos version 26.0 were used.

## 3. Results

### 3.1. Desciptive

**Figure 1:**
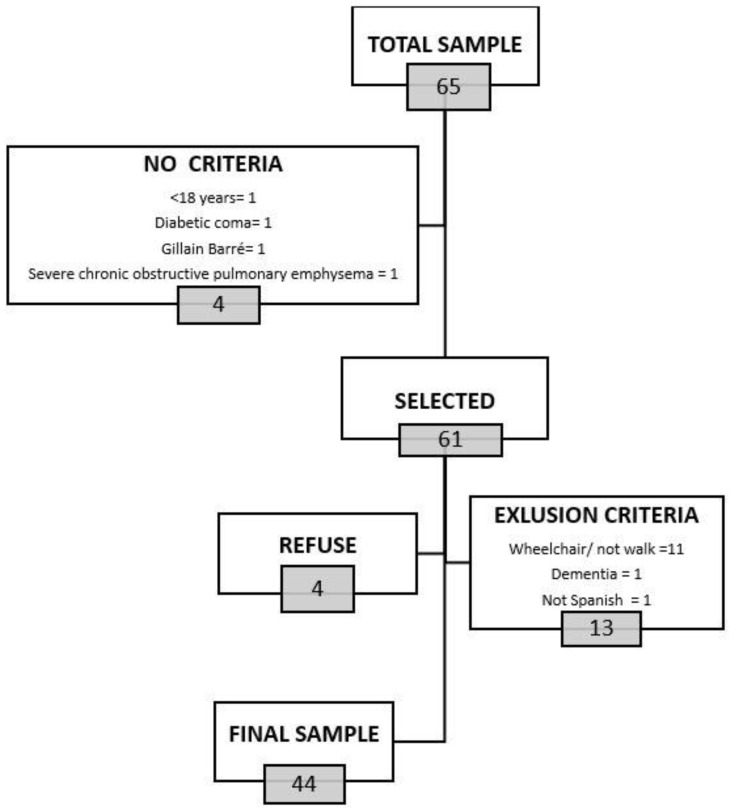
Flowchart.

The study population consisted of 65 subjects, 4 of whom did not meet the inclusion criteria and of the remaining 61, 4 did not wish to participate and 13 had one of the exclusion criteria (Figure 1).

A total of 44 subjects participated in this study, 26 men and 18 women, aged between 35 and 84 years, with a mean age of 65.35 years (SD 10.665), and with an evolution time since injury of between 2 months and 14 years (Mean: 4.12 years (SD 3.094). Socio- demographic data are presented in Table 1.

### 3.2. Validity

#### 3.2.1. Construct Validity

**Figure 2:**
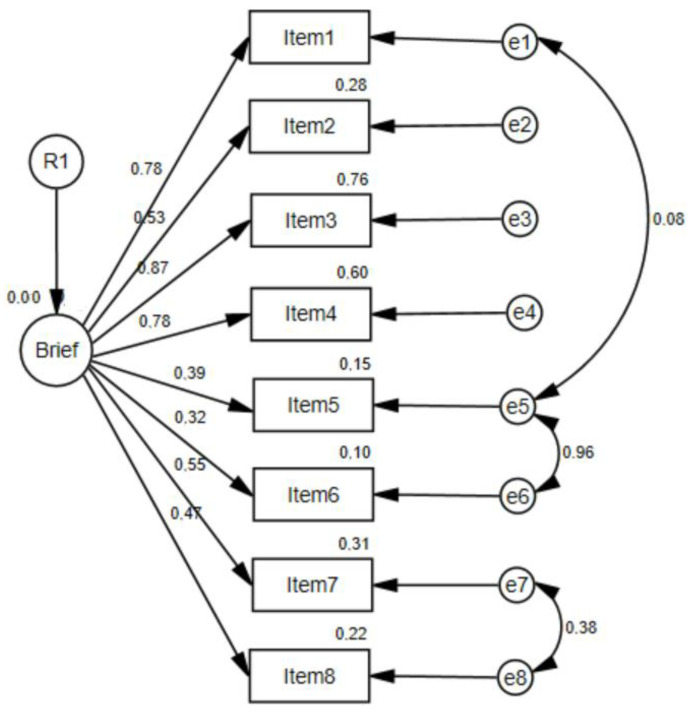
Confirmatory factor analysis of the Brief-BESTest.

To assess construct validity, a one-factor CFA was performed (Figure 2). To analyze the model fit, the S-B correction on the degrees of freedom, the Tucker Lewis Index (TLI), the Normalized Fit Index (NFI), the Comparative Fit Index (CFI) and the Root Mean Square Error of Approximation (RMSEA) values below 0.90 for CFI, TLI, and NFI and 0.08 for RMSEA were evaluated, indicating a good model fit (Table 2).

#### 3.2.2. Criterion Validity

Criterion validity was examined by correlating the total scores obtained in the Brief- BESTest with those obtained in the rest of the scales. The Spearman correlation coefficients between the different scales are shown in Table 3. There is a high positive correlation between the Brief-BESTest and the rest of the scales, except in the case of the Timed Up & Go Test where the correlation is moderate and negative.

The mean and standard deviation of the Brief-BESTest scores was 15.39 ± 5.41 points. The mean scores for the BESTest were 46.86 ± 10.41 points, those for the Mini-BESTest were 20.55 ± 5.72 points, and those for the Timed Up & Go Test were 12.38 ± 7.27 points.

#### 3.2.3. Predictive Validity

**Figure 3:**
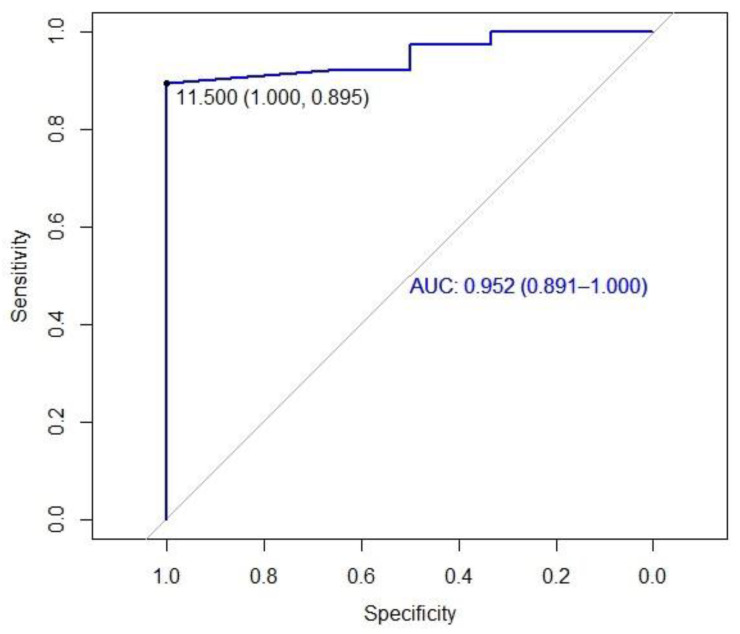
Receiver operating characteristic (ROC) plot of the Brief-BESTest scores using the Berg Balance Scale as reference to determine the cut-off point.

Through ROC analysis, the area under the curve (AUC), sensitivity, and specificity values were obtained (Figure 3). The cut-off point obtained for the Brief-BESTest was 11.5 with an area under the curve of 0.952 (0.891–1) (100% specificity and 89.5% sensitivity).

### 3.3. Reliability

The internal consistency shown by the Cronbach’s alpha coefficient value was 0.839 indicating a high internal consistency of the scale. Table 4 shows the corrected item-total correlation of the eight items. The corrected item-total correlation values are all positive and greater than 0.4. Cronbach’s alpha value did not increase if any of the items were deleted.

### 3.4. Floor Effect and Ceiling Effect

**Figure 4:**
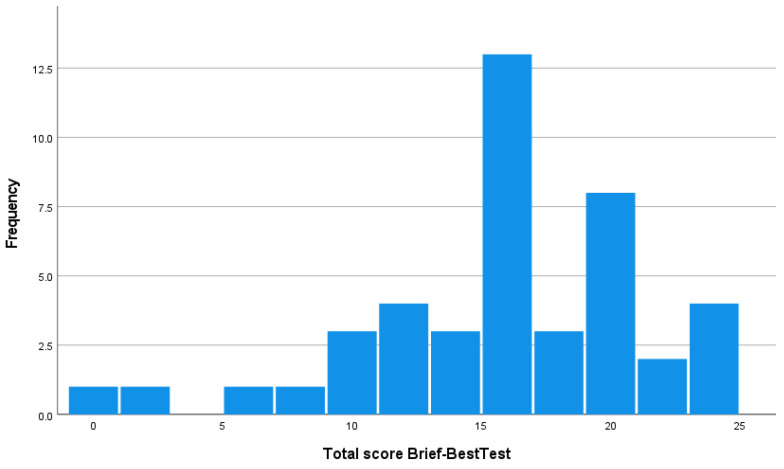
Distribution of Brief-BESTest scores.

Figure 4 shows the distribution of the Brief-BESTest scores. The Brief-BESTest scores showed moderate skewness (γ = −0.8). The proportion of participants scoring within the top 10% and bottom 10% of the possible range of Brief-BESTest values was 11.4% and 4.6%, respectively, indicating in this case that there is no substantial floor or ceiling effect.

## 4. Discussion

This study presents the validation of the Brief-BESTest balance scale and its psychometric properties. Balance assessment is a fundamental element in the rehabilitation of patients with ABI since postural alteration is one of the most frequent sequelae. This type of dysfunction generates imbalances in the patient’s weight bearing, which increases the risk of falls and makes difficult the performance of functional activities and activities of daily living reducing the social participation of these patients [21]. Detecting these alterations and knowing the patient’s evolution after treatment is essential to avoid these complications.

There are currently several validated scales with their cut-off points for determining the risk of falls in patients with ABI [22]. There are scales assessing gait speed and risk of falls, such as the TUG or the dynamic gait index [23], but these scales do not assess static aspects of balance. Other scales assess balance with all other aspects of function and mobility, such as the Fugl Meyer Assessment Scale [24] or the Modified Motor Assessment Scale Uppsala Akademiska Sjukhus [22], but they involve the assessment of less relevant aspects in the detection of balance status and increase the assessment time. The most commonly used specific balance scales are the BBS and the BESTest. The BBS scale is the most used in the literature [25], and a systematic review carried out in 2008 tested its psychometric properties in patients with stroke [25]; however, it has limitations as it does not perform an exhaustive control of all the systems involved in correct postural control. A study carried out in 2018 proved the test–retest reliability, construct validity, and responsiveness of the TUG and BBS for measuring balance in patients with chronic stroke. The reliability of the TUG (Intraclass Correlation Coefficient [ICC] = 0.98) and BBS (ICC = 0.99) were excellent. The standard error of measurement (SEM) of the TUG and BBS were 1.16 and 0.98, respectively. There was a significant correlation found between TUG and BBS (first reading [r] = −0.53) [23].

The BESTest overcomes this limitation and covers all aspects related to balance [8], but it is time-consuming, which makes it unsuitable for routine clinical practice. To address this limitation, a reduced scale was designed, which reduced the application time, the Mini-BESTest [26]. This scale again had the same limitations as the BBS scale since, although the application time is shorter, it does not consider all postural control systems and is less sensitive compared to other versions of the scale [27]. Furthermore, although it is lower than for other scales such as the BBS scale, it has a ceiling effect when detecting balance disturbances [28].

The Brief-BESTest scale has overcome these limitations and requires less application time, covers all domains, and has adequate psychometric properties [10]. Compared to the other versions of the BESTest, it is the fastest and easiest version to use while maintaining the necessary properties for the assessment of balance [12]. Specifically, it has 8 items, while the Mini-BESTest and the BESTest have 14 and 27 items, respectively, and the estimated application time for the Brief-BESTest is less than 10 min, compared to 15 min for the Mini-BESTest and the 30–40 min that the BESTest lasts. Moreover, according to the results of this study, it has been shown to have no ceiling or floor effect, making it a suitable tool for the assessment of balance and the detection of different needs in specific domains.

In the study by Padgett et al. [10], the findings suggest that all versions provide similar levels of accuracy in identifying the risk or not of falling in subjects with neuromuscular diseases, but provide initial support for the Brief-BESTest, which showed a reliability comparable to that of the Mini-BESTest and potentially higher sensitivity, while requiring half the items of the Mini-BESTest and representing all theoretical sections of the original BESTest.

Regarding interobserver reliability, the Brief-BESTest scale shows ICC values of 0.86 [12], 0.98 [10], and 0.90 [29], interpreted with good to excellent reliability. The Mini-BESTest and BESTest scales showed similar values, 0.84 and 0.86, respectively, in an observational study of 58 subjects [30]; according to a review of the BBS scale, its inter-rater reliability obtains a value of 0.97 [31]. A study on older adults confirmed the excellent results of inter-rater reliability and test–retest reliability of the four aforementioned scales, with inter-rater ICC values that ranged between 0.992–0.994 and a test–retest value of between 0.886–0.945 [27].

This scale has been translated into other languages such as Turkish [14]. The psychometric properties of the Turkish version were similar to those of the Spanish version, which had 44 subjects, and the Turkish version obtained similar values to those of the Spanish version. The reliability of the Turkish version yielded a Cronbach’s alpha of 0.881, slightly higher than the Spanish version of 0.839. No floor or ceiling effect were shown in both versions. Predictive validity was also tested, finding a cut-off point of 9 with an area under the curve of 0.872 (86% specificity and 80% sensitivity) for the Turkish version versus a cut-off point of 11 with an area under the curve of 0.952 (100% specificity and 89.5% sensitivity) for the Spanish version. Jacome et al. [32] placed the cut-off point at 16 points, as well as the work carried out by Huang et al. [11]. At the University of Hong Kong in patients with chronic stroke, the cut-off point was set below 18 points with an AUC of 0.942. Finally, the criterion validity was tested with the BBS in both versions, r = 0.732, *p* = 0.001 was obtained in the Turkish version, slightly higher than in the Spanish version r = 0.711, *p* < 0.001. According to this, Winairuk et al. [33] analyzed the criterion validity of the three versions of the BESTest scale, taking BBS as the gold standard, proving to be excellent for patients with subacute stroke, with correlation values of r = 0.96 between BBS and BESTest, r = 0.95 between BBS and Mini-BESTest, and r = 0.93 between BBS and Brief-BESTest.

Criterion validity has not been shown with the TUG. This difference has been observed in previous studies [34], in which the TUG classifies people with balance impairment differently from other specific scales for assessing balance. These differences may be due to the difficulty in determining the cut-off point of the TUG to indicate the risk of falls in different populations. In the case of the ABI population, the cut-off point was set at 15 s [22]. However, more recent validation studies determined that, in the chronic ABI population, the cut-off point was set at 13.49 s [35]. The mean score detected in our study population was 12.38 s, very close to the cut-off point. This situation hinders the possibility of properly detecting people with balance disturbances and the risk of falls, as values so close to the cut-off point, with differences of only one or two seconds, can complicate detection. This affects the criterion validity between the Brief-BESTest and the TUG.

It was not necessary to perform cross-cultural validation in this study because it was previously performed in the validation of the BESTest and Mini-BESTest scales [17].

The Brief-BESTest scale can determine the two areas that are collected on balance. On the one hand, anticipatory postural adjustments or the ability to prepare the body for disturbances in postural control that occur before certain actions, such as the adaptation of the body to move within a certain range, adjustments to achieve maximum stability limits, the sensory orientation required to maintain posture, movement in space during walking or the anticipatory postural adjustments themselves. All of these items refer to the anticipatory control of mobility and posture that allow for proper balance. It is important to detect the individual’s ability in these items because it is one of the most frequent alterations after ABI. It has been shown that there are delays in the activation of these mechanisms [36,37] and an incorrect activation of the musculature both in the order of muscle activation [38] and in the musculature that is activated [37] which determines that the functionality of the movement is neither adequate nor symmetrical [37]. The scale validated in this study assesses this ability in six of its items, determining possible deficits in the anticipatory postural adjustments of the person with ABI. On the other hand, reactive control to imbalance (items five and six) or the ability to react to unforeseen imbalances is assessed. People with ABI have been shown to present alterations in reactive control to imbalance, presenting an inability to perform the necessary adjustment measures [39] or increasing the latency time between imbalance and reactive action [40,41].

As it has been shown, people with ABI have limitations in both predictive and reactive systems, and according to previous studies, the relationship between these systems is moderate, so it seems that their activation depends on different neural systems [42]. For this reason, it is important that a balance scale assesses both types of abilities, to show a true state of balance and the patient’s risk of falling. The Brief-BESTest can perform this measurement and therefore seems to be suitable to give a fairly good idea of the state of all the systems that interfere with balance.

### 4.1. Limitations

This study has some limitations. The main limitation is determined by the relative heterogeneity of the sample, as patients with different stages of the disease were considered, including subacute and chronic patients. The alterations they present are similar, but it is possible that differences could be found if they were analyzed according to their time of evolution, so it would be possible to study in the future whether these differences exist. Another limitation is that the intra-class correlation coefficient for repeated measures is missing from the analysis, as well as inter-rater agreement and inter-rater reliability. Future studies will assess whether this scale is appropriate for other types of patients. Studies with more sample size are needed.

### 4.2. Clinical Implications

Having the Brief-BESTest tool in Spanish will allow for a quick and easy assessment of balance. It will be of great use in environments where healthcare workers have limited time to assess their patients.

## 5. Conclusions

The Spanish version of the Brief-BESTest scale is valid and reliable, showing adequate psychometric properties for the assessment of balance in stroke patients.

## Figures and Tables

**Table 1:** Socio-demographic data.

		Patients n = 44
Age * (years)		65.35 [62.03; 68.67]
Time since diagnosis * (years)		4.12 [3.16; 5.08]
Civil Status **	Single	3 (6.8)
	Married	35 (79.5)
	Divorced	3 (6.8)
	Widowed	3 (6.8)
Gender **	Male	26 (59.1)
	Female	18 (40.9)
Dominant side **	Right	42 (95.5)
	Left	2 (4.5)
Affected side **	Right	22 (50.0)
	Left	17 (38.6)
	Both	2 (4.5)
	None	1 (2.3)
Occupation **	Primary sector	2 (4.5)
	Secondary sector	4 (9.1)
	Service sector	30 (68.2)
	Others	7 (15.9)
Educational Level **	Primary	13 (29.5)
	Secondary	5 (11.4)
	High School	9 (20.5)
	University	15 (34.1)
	No studies	2 (4.5)

* Mean [95% Confidence Interval] ** number (percentage).

**Table 2:** Confirmatory factor analysis of the Brief-BESTest.

S-B$\chi^{2}/gl$	Goodness-of-Fit Test	TLI	NFI	CFI	RMSEA	AIC
1.12	$\chi^{2}=19.097;gl=17$	0.984	0.920	0.990	0.054	73.097

TLI (Tucker–Lewis index); NFI (normed fit index), CFI (comparative fit index); RMSEA (root mean square error of approximation), AIC (Akaike information criterion).

**Table 3:** Correlation coefficients between Brief-BESTest and BESTest, Mini-BESTest, Berg Balance Scale, and Timed Up & Go Test.

	BESTest	Mini-BESTest	BBS	TUG
Brief-BESTest total scores	r = 0.879	r = 0.808	r = 0.711	r = −0.404
	*p* < 0.001 **	*p* < 0.001 **	*p* < 0.001 **	*p* = 0.007

** *p* < 0.001.

**Table 4:** Corrected item-total correlation.

	Mean of Scale if Item Deleted	Scale Variance if Item Deleted	Corrected Item-Total Correlation	Squared Multiple Correlation	Cronbach’s Alpha if Item Deleted
Biomechanical Constraints	14.41	21.271	0.576	0.653	0.823
Stability limits/Verticality	13.23	25.110	0.586	0.379	0.824
Anticipatory Postural Adjustments (left)	14.16	21.579	0.717	0.661	0.801
Anticipatory Postural Adjustments (right)	14.30	22.353	0.624	0.538	0.813
Reactive Postural Control (left)	12.73	23.459	0.567	0.933	0.821
Reactive Postural Control (right)	12.75	24.006	0.497	0.930	0.829
Sensory Orientation	12.98	22.302	0.585	0.503	0.819
Stability in Gait	13.16	23.579	0.482	0.333	0.832

## Data Availability

All data will be available upon request to the corresponding author.
